# Persisting Muscle Dysfunction in Cushing’s Syndrome Despite Biochemical Remission

**DOI:** 10.1210/clinem/dgaa625

**Published:** 2020-09-03

**Authors:** Frederick Vogel, Leah T Braun, German Rubinstein, Stephanie Zopp, Heike Künzel, Finn Strasding, Adriana Albani, Anna Riester, Ralf Schmidmaier, Martin Bidlingmaier, Marcus Quinkler, Timo Deutschbein, Felix Beuschlein, Martin Reincke

**Affiliations:** 1 Department of Endocrinology, Medizinische Klinik und Poliklinik IV, LMU Klinikum, Ludwig Maximilians University Munich, München, Germany; 2 Endocrinology in Charlottenburg, Berlin, Germany; 3 Department of Internal Medicine I, Division of Endocrinology and Diabetes, University Hospital, University of Würzburg, Würzburg, Germany; 4 Klinik für Endokrinologie, Diabetologie und Klinische Ernährung, UniversitätsSpital Zürich, Zürich, Switzerland

**Keywords:** muscle, cortisol, ACTH, sarcopenia, hypercortisolism, diabetes

## Abstract

**Context:**

Glucocorticoid-induced myopathy is a characteristic symptom of endogenous Cushing’s syndrome (CS). Its long-term outcome is largely unknown.

**Objective:**

To evaluate long-term muscle function following the remission of endogenous CS.

**Study Design:**

Observational longitudinal cohort study.

**Setting:**

Tertiary care hospitals and a specialized outpatient clinic.

**Patients:**

As part of the prospective multicenter German Cushing’s Registry, we assessed muscle strength in patients with overt endogenous CS. We studied the patients at the time of diagnosis (n = 88), after 6 months (n = 69), and thereafter annually, following surgical remission over a period of up to 4 years (1 year: n = 55; 2 years: n = 34; 3 years: n = 29; 4 years: n = 22). Muscle function was evaluated by hand grip strength and by chair rising test.

**Results:**

Grip strength was decreased to 83% of normal controls (100%) at the time of diagnosis. It further decreased to 71% after 6 months in remission (*P *≤ 0.001) and showed no improvement during further follow-up compared with baseline. Chair rising test performance improved initially (8 seconds at baseline vs 7 seconds after 6 months, *P* = 0.004) but remained at this reduced level thereafter (7 seconds after 3 years vs 5 seconds in controls, *P* = 0.038). In multivariate analysis, we identified, as predictors for long-term muscle dysfunction, age, waist-to-hip ratio, and hemoglobin A1c at baseline. Furthermore, muscle strength during follow-up was strongly correlated with quality of life.

**Conclusion:**

This study shows that CS-associated myopathy does not spontaneously resolve during remission. This calls for action to identify effective interventions to improve muscle dysfunction in this setting.

Glucocorticoid-induced myopathy with self-reported muscle weakness is present in up to 60% of patients with florid Cushing’s syndrome (CS). It is reported to be more frequent in men than in women (1, 2). The development of proximal muscle wasting and weakness is also a typical side effect of systemic glucocorticoid treatment. Based on the common use of steroids for the treatment of several medical conditions, exogenous glucocorticoids have become the most common reason for drug-induced myopathy (3).

Cushing’s syndrome-associated myopathy particularly affects the proximal part of the limbs (4). The clinical management of glucocorticoid-induced myopathy is difficult, as patients typically experience relevant muscular impairment at the time of initial diagnosis. Quantitative muscle ultrasound is proposed to be a useful diagnostic tool for the detection of CS before the development of symptoms (5). A specific therapy is not available so far, and current treatment recommendations consist of adequate protein intake and moderate physical exercise (6–9). Given that endogenous CS is a rare disease, so far, associated myopathy has been studied only in small patient cohorts. Several characteristic clinical features of CS, including cognitive impairments, fatigue, and an increased cardiovascular risk, can persist even years after a successful cure (10, 11). We and others have suggested that CS-associated myopathy and muscle damage may continue in the early recovery phase after successful treatment (12–14), but its long-term prognosis and outcome is unknown. In retrospective, cross-sectional studies, patients in long-term remission showed decreased muscle strength and a lower aerobic exercise capacity compared to controls (14, 15). Whether this finding is due to a long-term change of muscle fibers in terms of an irreversible myopathy or due to a persisting cardiorespiratory dysfunction remains controversial. To analyze the long-term outcome of muscle dysfunction in CS, we evaluated the prospective data of the German Cushing’s Registry.

## Patients and Methods

### Patients

The study was performed as part of the German Cushing’s Registry, a multicenter longitudinal cohort study, which has enrolled a total of 317 patients in 6 centers since 2012. For the current prospective study, we selected 88 patients with endogenous CS and consecutive muscle function tests diagnosed between 2012 and 2018 at 3 sites of the German Cushing’s Registry (Ludwig Maximilians University Munich, n = 66; University Hospital Würzburg, n = 18; Endocrinology outpatient clinic in Charlottenburg, Berlin, n = 4). All patients had typical signs and symptoms of CS. The diagnosis and subtype differentiation of CS were done as reported earlier according to current guidelines and recommendations (14, 16). All 88 patients (49 with Cushing’s disease, 34 patients with cortisol-producing adrenal adenoma, and 5 patients with ectopic CS) were followed-up prospectively. Patients with subclinical hypercortisolism, adrenocortical cancer, and adrenostatic or radiotherapeutic therapy were excluded. All 88 patients underwent successful surgery, leading to sustained biochemical remission. In 9 cases with pituitary CS, a second surgery was necessary to achieve remission. Median time to remission from diagnosis was 2 months, and median time to remission from the first symptoms was 25 months. Due to pre-existing rheumatic disorders or degenerative joint diseases (ie, coxarthrosis), 13 patients could not perform either grip strength or the chair rising test (CRT). Out of all 88 patients included with baseline muscle function examination, grip strength data was available from 85 patients and CRT data from 78 patients. Patients were re-examined clinically and biochemically in regular follow-up visits 6 and 12 months after final surgery, and afterwards annually. Due to a shorter remission time of recently included patients, the number of patients decreases with time (6 months: n = 69; 1 year: n = 55; 2 years: n = 34; 3 years: n = 29; 4 years: n = 22). The mean follow-up for all patients with CS was 2.2 years. Only patients with at least 1 follow-up visit in addition to baseline visit were included in the study. For the comparison of muscle function, we recruited a control group of rule-out CS patients who had no biochemical evidence of CS. The control group (n = 29) was matched according to body mass index (BMI), age, and gender of patients with CS who have been in remission for 3 years (n = 29; Supplement Table 1; all supplementary material and figures are located in a digital research materials repository (17)). All patients and controls gave written, informed consent. The protocol was approved by the ethics committee of the participating centers.

### Muscle strength measurements

Hand grip strength was measured 3 times on both hands per visit in a sitting position. The measurements were performed in a standardized manner with the JAMAR hydraulic hand dynamometer (Patterson Medical, Nottinghamshire, UK). The mean grip strengths (kg) for the dominant and nondominant hand were calculated out of 3 repetitions. The hand with the better performance at baseline was defined as the dominant hand. To adjust for age and gender, grip strength was standardized (normalized grip strength) to the manufacturer’s information on normative grip strength data (18, 19). Mean normalized grip strength was determined from the mean value of dominant and nondominant grip strength according to age and gender. To determine muscle function of the lower limbs, CRT was performed, as previously described in other studies (14, 20). The test was conducted by rising up from a sitting position 5 times as fast as possible. The patients kept their arms crossed over their chest while performing the exercise. The time (seconds) needed for the procedure (with ending in a standing position) was recorded and compared.

### Bioimpedance measurements and biometrics

By using a bioimpedance measuring device at 50 kHz with 400 µA by Data Input (Poecking, Germany), body cell mass and body fat percentage was estimated according to the manufacturer’s information. Two pairs of current-introducing and voltage-sensing electrodes were attached to the dorsum of a hand and a foot. All impedance measurements were taken after fasting, the arms relaxed at the sides without touching the body. Bioimpedance and anthropometric measurements such as BMI and waist-to-hip ratio (WHR) were performed by the same skilled investigator in a standardized manner.

### Quality of life

To analyze quality of life in patients with CS, 2 inventories were used: the disease-specific questionnaire, Cushing’s quality of life (CushingQoL) (21), and the Short Form 36 health survey (SF-36) (22). The SF-36 Physical Component Summary and SF-36 Mental Component Summary were gained by weighting and summing up the original scales of the SF-36 according to Ellert et al (23), and in relation to a German normative population (24).

### Laboratory analysis

In all patients, blood samples were taken in a fasting state at time of diagnosis, 6 months after successful surgery and in line with the follow-up visits once a year. The analyses were performed in the central laboratories of the involved centers using standard methods.

### Statistical evaluation

Statistical analysis was performed using SPSS (version 25). Patient characteristics are shown as median and 25^th^ and 75^th^ percentiles in brackets. For comparison between different time points, the Wilcoxon signed rank test was used. Differences between the groups were analyzed using the Mann-Whitney U test and Kruskal–Wallis test. Based on a difference of 23% in normalized grip strength and 2 seconds in CRT after 3 years, a power of 0.8 and *P*-level of 0.05, the study required the participation of a minimum of 18 patients per group. Because of the decreasing number (<20) of patients with CS after 5 years, we limited the observation period to 4 years. To identify independent variables influencing muscle strength, outcome correlations between muscle strength and clinical parameters were determined using the Spearman’s rank correlation coefficient. Multiple linear regression analysis was performed with the variables of age, WHR, and hemoglobin A1c (HbA1c). *P*-values of <0.05 were considered to indicate statistical significance.

## Results

### Patient characteristics

Table 1 shows the clinical and biochemical characteristics of the included patients with CS. Due to pre-existing rheumatic diseases or chronic degenerative arthropathies, 3 and 10 patients, respectively, could not participate in grip strength measurements or CRT. Six months after successful surgery, we observed the expected reduction in BMI (*P *≤ 0.001), WHR (*P *≤ 0.001 in females, *P* = 0.008 in males), and HbA1c (*P *≤ 0.001; Table 1).

**Table 1.** Baseline and 6-month follow-up characteristics of all patients with Cushing’s syndrome

Patient Characteristics	Reference Interval	n	Baseline	6-month Remission	*P*-value Baseline vs 6-month Remission
Sex, female/male	–	88	69 (78%) /19 (22%)	–	–
Age, years	–	88	49 [40; 59]	–	–
Diagnosis, pituitary/adrenal/ectopic	–	88	49 (56%)/34 (38%)/5 (6%)	–	–
Time to remission from diagnosis, months	–	88	2 [1; 5]	–	–
Time to remission from first symptoms, months	–	88	25 [12; 74]	–	–
Pre-existing diabetes mellitus, yes/no	–	88	22 (25%)/66 (75%)	–	–
Bone density, T-score	–	46	-1.6 [-2.0; -0.9]	–	–
BMI, kg/m^2^	–	88	29 [25; 33]	27 [24; 31]	**≤0.001**
Waist-to-hip ratio, female	<0.85	61	0.94 [0.87; 1.02]	0.91 [0.82; 0.96]	**≤0.001**
Waist-to-hip ratio, male	<0.90	19	1.04 [1.00; 1.12]	0.93 [0.89; 0.98]	**0.008**
HbA1c, %	–	86	5.9 [5.4; 6.4]	5.5 [5.2; 6.1]	**≤0.001**
HOMA-Index	–	72	3.3 [2.0; 5.0]	2.4 [1.1; 4.7]	0.341
UFC, µg/24h	≤83.0	86	366 [196; 708]	35 [7; 61]	**≤0.001**
LNSC, ng/mL	≤1.5	85	7.2 [3.5; 11.9]	0.9 [0.4; 1.7]	**≤0.001**
DST, 1 mg	≤2.0	86	14.2 [7.1; 23.5]	1.1 [1.0; 1.7]	**≤0.001**
ACTH in pituitary CS, pg/mL	4–50	49	54 [30; 91]	11 [5; 19]	**≤0.001**
ACTH in adrenal CS, pg/mL	4–50	34	4 [2; 5]	9 [5; 20]	**≤0.001**
ACTH in ectopic CS, pg/mL	4–50	5	103 [66; 253]	11 [4; 23]	**0.043**

Data are given as median and 25^th^ and 75^th^ percentile in brackets. Comparisons were performed by a Wilcoxon signed rank test. Bold *P*-values indicate statistical significance.

Abbreviations: ACTH, adrenocorticotropic hormone; BMI, body mass index; CS, Cushing’s syndrome; DST, dexamethasone suppression test; HbA1c, hemoglobin A1c; HOMA, Homeostasis Model Assessment; LNSC, late night salivary cortisol; UFC, urinary free cortisol.

### Grip strength temporarily worsens during steroid withdrawal and remains impaired in long-term follow-up

**Figure 1.**
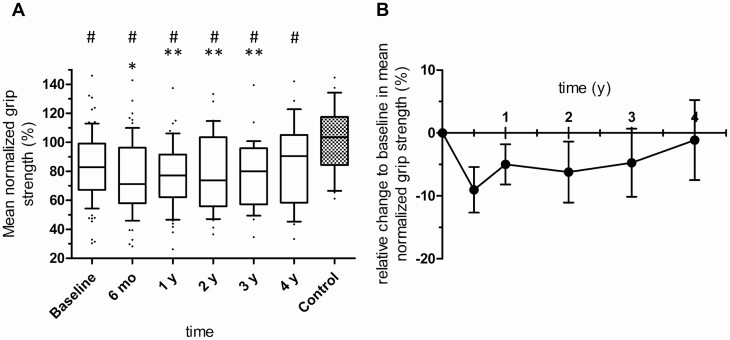
**A:** Age- and gender-corrected grip strength (%) in patients with Cushing’s syndrome (CS) at baseline and after successful surgery. Box and whiskers (10^th^ to 90^th^ percentile). Baseline: n = 85; 6 months (mo): n = 69; 1 year (y): n = 55; 2 y: n = 34; 3 y: n = 29; 4 y: n = 22; control: n = 29. Comparisons over time were performed by a Wilcoxon signed rank test and comparisons with control by a Mann-Whitney U test. *P *< 0.05 was considered statistically significant; *P < 0.05 vs baseline, **  *P *< 0.05 vs 6-mo follow-up, # *P *< 0.05 vs control; the higher percentage indicates greater muscle strength. **B:** Relative changes to baseline in mean normalized grip strength (%) of all patients with CS in remission. Data are given as mean ± SEM.

Mean age- and gender-corrected grip strength of the dominant and nondominant hand (mean normalized grip strength) decreased from 83% at diagnosis to 71% 6 months after successful treatment (*P *≤ 0.001 vs baseline, Fig. 1A and 1B). One year after surgery, mean normalized grip strength had slightly increased to 77% (*P* = 0.036 vs 6 months) and remained stable at this reduced level without further improvement during follow-up until the end of the observation period at 4 years (*P* = 0.030 at 4 years follow-up vs controls, Fig. 1A).

### Chair rising test performance initially improves but remains impaired during long-term follow-up

**Figure 2.**
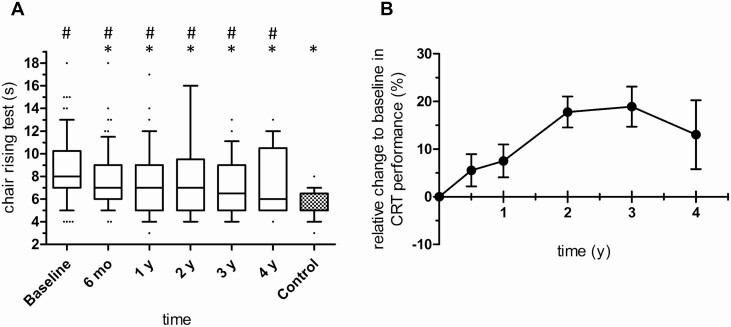
**A:** Chair rising test performance (seconds) of patients with Cushing’s syndrome (CS) at baseline and after successful surgery. Box and whiskers (10^th^ to 90^th^ percentile). Baseline: n = 78; 6 months (mo): n = 64; 1 year (y): n = 50; 2 y: n = 29; 3 y: n = 28; 4 y: n = 21; control: n = 29. Comparisons over time were performed by a Wilcoxon signed rank test and comparisons with control by a Mann-Whitney U test. *P *< 0.05 was considered statistically significant; *  *P *< 0.05 vs baseline, # *P *< 0.05 vs control. Shorter time (seconds) indicates greater muscle strength. **B:** Relative changes to baseline in chair rising test performance (%) of all patients with CS in remission. Data are given as mean ± SEM.

Patients with CS in remission showed improved CRT performance 6 months after successful surgery compared with baseline (median 8 seconds vs 7 seconds at 6 months follow-up, *P* = 0.004, Fig. 2A and 2B). No further improvement was observed over the next 4 years. Compared with controls, CRT remained abnormal over time (7 seconds in CS at 3 years follow-up vs 5 seconds in controls, *P* = 0.038, Fig. 2A).

### Factors influencing long-term outcome

We identified age, WHR, and HbA1c at the time of diagnosis as associated with the long-term muscle function outcome at 3 years in univariate and multivariate analysis (Tables 2 and 3 and Supplement Fig. 1; all supplementary material and figures are located in a digital research materials repository (17)). Consistent with these findings, a subgroup analysis of patients with pre-existing diabetes mellitus revealed a poorer CRT performance at 3 years (*P* = 0.009 vs patients without pre-existing diabetes mellitus), whereas Cushing subtype or duration of CS manifestation prior to surgery had no significant influence (Table 2). In a subgroup analysis of 29 patients followed for more than 3 years, patients with long-term glucocorticoid replacement therapy (n = 16) showed similar muscle strength compared with patients without glucocorticoid replacement (n = 13). Urinary free cortisol, late night salivary cortisol, and 1 mg dexamethasone suppression test at diagnosis did not correlate with muscle strength outcomes. Moreover, quality of life, BMI, estimated muscle mass, and body fat percentage at baseline were not associated with muscle strength outcome (Supplement Table 2; all supplementary material and figures are located in a digital research materials repository (17)).

**Table 2.** Correlation analysis of predictor variables at baseline and long-term muscle function after 3 years in remission of Cushing’s syndrome

Predictor Variable	Muscle Function	n	*P*-value	Spearman’s Coefficient
Age	GS	29	0.934	0.019
	CRT	28	**≤0.001**	**0.598**
WHR	GS	29	**0.014**	**-0.514**
	CRT	28	**0.010**	**0.478**
HbA1c	GS	29	0.495	-0.153
	CRT	28	**0.009**	**0.485**
Time to remission from first symptoms	GS	29	0.474	0.138
	CRT	28	0.214	-0.242
Subtype, pituitary vs adrenal vs ectopic	GS	20 vs 8 vs 1	0.794	–
	CRT	20 vs 7 vs 1	0.428	–
Pre-existing diabetes mellitus, yes vs no	GS	10 vs 19	0.910	–
	CRT	9 vs 19	**0.009**	–

Decreasing values of GS corresponds to increased upper limb myopathy; increasing values of CRT corresponds to increasing lower limb myopathy. Correlations were performed by a Spearman’s correlation analysis. Comparisons were performed by a Mann-Whitney U test, and for more than 2 groups by a Kruskal–Wallis test. Bold *P*-values indicates statistical significance.

Abbreviations: CRT, chair rising test; GS, grip strength; HbA1c, hemoglobin A1c; WHR, waist-to-hip ratio; y, year.

**Table 3.** Results of multivariate linear regression model of muscle strength measurements 3 years after treatment and the predictor variables of age, waist-to-hip ratio, and HbA1c

Dependent Variable	Predictor Variables	Standardized Coefficients			
		β	*P-*value	R^2^	*P-*value
CRT performance				0.288	**0.011**
	Age	0.469	**0.031**		
	WHR	0.093	0.655		
	HbA1c	0.124	0.584		
Normalized grip strength				0.308	0.083
	Age	0.173	0.479		
	WHR	-0.604	**0.016**		
	HbA1c	0.067	0.801		

Bold *P*-values indicate statistical significance.

Abbreviations: β, beta; CRT, chair rising test; HbA1c, hemoglobin A1c; WHR, waist-to-hip ratio.

### Changes of body composition

Anthropometric measurements showed the expected fast decrease in BMI and WHR after successful surgery (Table 1). By performing bioimpedance measurements, body fat percentage and body cell mass (as surrogates for muscle mass) could be estimated. Concerning estimated muscle mass, no relevant differences were observed between baseline and follow-up assessments (Supplement Fig. 2; all supplementary material and figures are located in a digital research materials repository (17)). At baseline, estimated muscle mass of 25 kg correlated with bone density (median T-score -1.6, *P* = 0.017). Body fat percentage decreased from 35% at baseline to 33% at 6 months (*P* = 0.045) and 29% at 1-year follow-up (*P* = 0.003, Supplement Fig. 2; all supplementary material and figures are located in a digital research materials repository (17)).

### Muscle function is associated with quality of life

Quality of life improved over time according to both the CushingQoL and SF-36 questionnaires (Supplement Table 3; all supplementary material and figures are located in a digital research materials repository (17)). Spearman’s correlation analysis showed a strong correlation between muscle function and quality of life during follow-up (Table 4). These data suggest that impaired quality of life not only depends on the severity of muscle dysfunction during hypercortisolism but also on the myopathy outcome in patients with CS in remission.

**Table 4.** Correlation between muscle function outcome and quality of life in patients with Cushing’s syndrome in remission

Time Points	Muscle Function	QoL Questionnaire	n	*P*-value	Spearman’s Coefficient
Baseline	CRT	CushingQoL	53	**0.030**	**-0.298**
		SF-36 PCS	57	**0.002**	**-0.410**
6 months	CRT	CushingQoL	39	**0.042**	**-0.327**
		SF-36 PCS	42	**0.007**	**-0.413**
1 y	CRT	SF-36 PCS	30	**≤0.001**	**-0.626**
2 y	CRT	CushingQoL	21	**≤0.001**	**-0.714**
		SF-36 PCS	22	**≤0.001**	**-0.668**
		SF-36 MCS	22	**0.013**	**-0.522**
3 y	Grip strength	CushingQoL	17	**0.050**	**0.480**
4 y	Grip strength	CushingQoL	10	**0.029**	**0.685**
	CRT	CushingQoL	10	**0.010**	**-0.766**
		SF-36 PCS	11	**≤0.001**	**-0.834**
		SF-36 MCS	11	**0.010**	**-0.737**

Baseline and follow-up assessments before and after successful treatment. Worse muscle function in CRT corresponds to higher readings, while worse muscle function in grip strength corresponds to lower readings. Correlations were performed by Spearman’s correlation analysis. Bold *P*-values indicate statistical significance.

Abbreviations: CRT, chair rising test; CushingQoL, Cushing’s quality of life; QoL, quality of life; SF-36 MCS = Short Form 36 Mental Component Summary; SF-36 PCS, Short Form 36 Physical Component Summary; y, year.

## Discussion

This is the first prospective longitudinal study investigating the long-term outcome of CS-associated myopathy. The main finding of our study is that muscle strength remains impaired even after years in remission, and independent of glucocorticoid replacement status. Another interesting finding is that at 6 months follow-up, grip strength and CRT performance show opposite effects; whereas grip strength has worsened, CRT performance has improved. Because of a correlation between CRT performance and BMI at baseline (*P* = 0.007), it is likely that an improvement in CRT after achieving remission is due to the considerable reduction in BMI. However, grip strength remained impaired and showed no improvement during follow-up, while the temporary worsening of grip strength during the steroid withdrawal phase seems to be reversible. Despite biochemical remission, myopathy can thus be regarded as another persistent clinical symptom, as it is known for cognitive function, mental fatigue, and cardiovascular risk (10, 11, 25). Why patients with CS in remission showed a temporary worsening in grip strength 6 months after surgery remains unclear in term of pathophysiology. That muscle function and thus myopathy is affected by glucocorticoid withdrawal due to a relative glucocorticoid resistance or inflammatory processes is quite conceivable (26, 27). Also, high-dose glucocorticoid replacement for the prevention of a steroid withdrawal syndrome directly after surgery could account for the transient aggravation. Grip strength measurements performed by the JAMAR hydraulic hand dynamometer depends on muscle function but also requires an intact function of joints and connective tissue. It is possible that muscle and joint pain, which can as well be provoked by glucocorticoid withdrawal, affect strength measurements.

As predictors for myopathy outcome and muscle function in remission of CS, we identified age, WHR, and HbA1c. Age correlated inversely with relative changes of grip strength and CRT in relation to baseline (Supplement Fig. 1; all supplementary material and figures are located in a digital research materials repository (17)). Moreover, a high WHR and HbA1c at baseline were associated with a poorer outcome in muscle function (Tables 2 and 3), while BMI and body fat percentage estimated with bioimpedance measurements were not. These results suggest that particularly an unfavorable fat distribution with elevated WHR and diabetic metabolic state contributes to adverse outcome of myopathy. These findings are concordant with our previous analysis of patients with florid CS and matched obese controls, where we found that patients suffering from both hypercortisolism and impaired fasting glucose had the strongest impairment in grip strength and CRT performance, implicating impaired glucose metabolism as an important driver in the pathomechanism of myopathy. According to our data analysis we assume that impaired glucose metabolism affects both muscle function during hypercortisolism and the long-term outcome of CS-associated myopathy. The link between impaired muscle function and diabetes has already been described, but the underlying pathomechanisms remain largely unclear (28). Furthermore, other factors such as growth hormone and sex hormone suppression are proposed to play a crucial role in influencing CS-associated myopathy in the long run (29, 30).

Whether the necessity of a long-term glucocorticoid replacement influences muscle strength or myopathy outcome remains controversial. Geer et al found an inverse correlation between duration of glucocorticoid replacement and skeletal muscle mass in patients with CS in remission, suggesting continued muscle loss resulting from glucocorticoid replacement therapy (13). In our subgroup analysis of 29 patients followed for more than 3 years in remission, we could not observe any differences in muscle function or estimated muscle mass by bioimpedance measurements between patients with or without glucocorticoid replacement 3 years after surgical cure. Consistent with previous findings on the body composition of patients with CS in remission (12), estimated body fat percentage decreased, whereas estimated muscle mass remained at a constant level. These results indicate that weight loss and reduction of BMI after successful treatment is most likely due to a loss of body fat than due to continuing muscle loss after hypercortisolism. However, clinically manifest—even though subclinical—hypercortisolism was associated with muscle waste and the severity of hypercortisolism was correlated with lower muscle mass (31, 32), the remission of CS exhibited no relevant gain in muscle function or muscle mass (12, 13). The severity of cortisol excess in patients with adrenal CS was also reported to be related to visceral fat area, visceral-to-subcutaneous fat ratio and skeletal muscle radiodensity 1 year after adrenalectomy (33). In a recently published cross-sectional study with 17 patients, Roerink et al reported a reduced aerobic exercise capacity after years in remission. Due to nonsignificant differences in muscle fiber cross-sectional area and capillarization, impaired aerobic capacity was explained by a persisting cardiac dysfunction, as seen in patients with florid CS (15). In terms of pathology, muscle biopsy in glucocorticoid-induced myopathy demonstrated severe histochemical type-2 muscle fiber atrophy (34). Whether these alterations during hypercortisolism are reversible and whether intermuscular fat infiltration plays a critical role in its pathophysiology remains largely unknown (35). Likewise, if muscle atrophy leads to an abnormal glucose metabolism or if a diabetic metabolic state promotes atrophy and/or fat infiltration is uncertain. We have recently shown that hand grip strength seems to be more susceptible to hypercortisolism in a glucocorticoid receptor A3669G wild type than in A3669G minor allele carriers, explaining the partially interindividual differences of glucocorticoid-induced myopathy in patients with endogenous CS (36). Given the fact that corticosteroid-induced myopathy also affects 60% of patients receiving corticosteroid treatment (3), and muscle function showed a strong correlation with quality of life, especially in the long-term period, there is great importance to clarify pathophysiologic basics and to find treatment options for patients with glucocorticoid-induced myopathy.

### Strengths and limitations of the study

This is a prospective study with a reasonable number of patients having adequate statistically power. A limitation of the study is the decreasing number of patients with time in remission caused by the continuing longitudinal inclusion of patients with florid CS between 2012 and 2018. Given that the statistical power of this study was calculated based on grip strength and CRT changes, it cannot be ruled out that the statistical power for other results and clinical parameters is insufficient. Some results might therefore change after all 88 patients have completed the 4-year follow-up. Moreover, a further limitation could be a selection or observer bias, as the study was performed in 3 sites of the German Cushing’s Registry.

## Conclusion

Muscle function of patients with CS in remission remained impaired in the long term. Influencing factors for myopathy outcome are age, WHR, and HbA1c, suggesting that a consistent and strict treatment of diabetic metabolic state during hypercortisolism is mandatory. Further studies are necessary to understand pathophysiology and to develop effective treatment options.

## Data Availability

All data generated or analyzed during this study are included in this published article or in the data repositories listed in References.

## Additional Information

***Disclosure Summary:*** The authors have nothing to disclose.
